# Profiling the peripheral immune response to ex vivo TNF stimulation in untreated juvenile idiopathic arthritis using single cell RNA sequencing

**DOI:** 10.1186/s12969-023-00787-x

**Published:** 2023-02-15

**Authors:** Kathleen J. Imbach, Nicole J. Treadway, Vaishali Prahalad, Astrid Kosters, Dalia Arafat, Meixue Duan, Talia Gergely, Lori A. Ponder, Shanmuganathan Chandrakasan, Eliver E. B. Ghosn, Sampath Prahalad, Greg Gibson

**Affiliations:** 1 Center for Integrative Genomics, School of Biological Sciences, Georgia Institute of Technology, Atlanta, GA 30332 USA; 2 Department of Pediatrics, Emory University School of Medicine, Atlanta, GA 30223 USA; 3 Lowance Center for Human Immunology, Division of Immunology, Department of Medicine, Emory University School of Medicine, Atlanta, GA 30223 USA; 4 Center for Immunity and Applied Genomics, Children’s Healthcare of Atlanta, Atlanta, GA 30223 USA; 5 Aflac Cancer and Blood Disorders Center, Department of Pediatrics, Children’s Healthcare of Atlanta, Emory University School of Medicine, Atlanta, GA 30223 USA; 6 Department of Human Genetics, Emory University School of Medicine, Atlanta, GA 30223 USA

**Keywords:** JIA, Transcriptomics, Immunoprofiling, scRNAseq

## Abstract

**Background:**

Juvenile Idiopathic Arthritis (JIA) is an autoimmune disease with a heterogenous clinical presentation and unpredictable response to available therapies. This personalized transcriptomics study sought proof-of-concept for single-cell RNA sequencing to characterize patient-specific immune profiles.

**Methods:**

Whole blood samples from six untreated children, newly diagnosed with JIA, and two healthy controls were cultured for 24 h with or without ex vivo TNF stimulation and subjected to scRNAseq to examine cellular populations and transcript expression in PBMCs. A novel analytical pipeline, *scPool*, was developed wherein cells are first pooled into pseudocells prior to expression analysis, facilitating variance partitioning of the effects of TNF stimulus, JIA disease status, and individual donor.

**Results:**

Seventeen robust immune cell-types were identified, the abundance of which was significantly affected by TNF stimulus, which resulted in notable elevation of memory CD8 + T-cells and NK56 cells, but down-regulation of naïve B-cell proportions. Memory CD8 + and CD4 + T-cells were also both reduced in the JIA cases relative to two controls. Significant differential expression responses to TNF stimulus were also characterized, with monocytes showing more transcriptional shifts than T-lymphocyte subsets, while the B-cell response was more limited. We also show that donor variability exceeds the small degree of possible intrinsic differentiation between JIA and control profiles. An incidental finding of interest was association of HLA-DQA2 and HLA-DRB5 expression with JIA status.

**Conclusions:**

These results support the development of personalized immune-profiling combined with *ex-vivo* immune stimulation for evaluation of patient-specific modes of immune cell activity in autoimmune rheumatic disease.

**Supplementary Information:**

The online version contains supplementary material available at 10.1186/s12969-023-00787-x.

## Introduction

Personalized genomic medicine is predicated on the notion that patients possess individualized profiles of gene activity that predispose them to specific trajectories of disease progression, and may indicate bespoke therapeutic regimes [1, 2]. While polygenic risk scores are widely being considered for stratification of risk of disease onset [3], these cannot capture the dynamic nature of pathology during flares or associated with treatment, so transcriptome profiling of relevant cell types is emerging as a complementary genomic strategy. For example, in systemic lupus erythematosus, patients have been grouped into a dozen categories of peripheral blood immune cell proportions and gene expression that correlate with disease activity [4]. Whether or not these groups respond differently to therapeutic intervention remains to be evaluated. Single cell RNA sequencing provides enhanced resolution over bulk analysis [5], facilitates direct comparison of diverse cell types and states, and is expected to provide critical linkages between genome-wide association studies and understanding of pathological gene function [6].

Juvenile Idiopathic Arthritis (JIA) is characterized by chronic (> 6 weeks) inflammatory synovitis of unknown etiology in children under 16. The International League of Associations for Rheumatology (ILAR) has identified seven JIA categories including oligoarthritis, polyarthritis rheumatoid factor (RF)-negative, polyarthritis RF-positive, systemic, psoriatic, enthesitis-related, and undifferentiated arthritis [7]. These designations, meant to delineate homogenous, non-overlapping subgroups of disease, are based primarily on clinical features including number of joints involved, presence of systemic symptoms, results of laboratory values such as RF, and family history of conditions like psoriasis. Importantly, the extent to which these distinguishing signs and symptoms for JIA categories map on to distinct pathophysiologies is unclear. Although genomic associations among ILAR categories of JIA offer evidence that some human leukocyte antigen (HLA) variants do associate with subtypes of disease [8] and distinct bulk PBMC gene expression profiles are associated with subtypes of JIA [9], significant clinical and molecular heterogeneity within is also apparent.

Currently, at onset, physicians cannot predict the clinical course of disease regarding severity or symptom duration. Furthermore, subtypes of disease do not respond homogenously to treatment options including NSAIDs, corticosteroids, disease modifying anti-rheumatic drugs (DMARDs), and biologics. On a molecular level, microarray analysis of peripheral whole blood distinguished three subsets of polyarticular arthritis defined by gene expression signatures [10], but age of onset also influences these signatures [11] and expansive single cell profiling is needed to help interpret the sources of variability in treatment response. Combining flow cytometry techniques with immuno-transcriptomics has helped investigators distinguish cases with JIA from healthy controls as well as other systemic diseases [12–15]; aid with prognosis of JIA [16–18]; and predict and monitor response to treatment for inflammatory arthritis [19–23].

A recent application of droplet-based single cell RNA sequencing suggests that synovial innate lymphoid cells may be pathogenic in oligoarticular and polyarticular, RF negative JIA [24]. Related studies of adult rheumatoid arthritis (RA) have noted differences in T_helper_ and HLA-DR + CD27- cytotoxic T-cell activity in the synovium [25], while also characterizing excessive myeloid activation in ACPA-negative cases [26]. Frequent longitudinal profiling of peripheral blood led Orange et al., to identify an inflammatory signature of flares, which may be preceded 2 weeks earlier by B-cell activation and thence fibroblast priming [27].

Here we report a proof-of-principle study of scRNAseq analysis of the effect of PBMC activation by 24-h Tumor Necrosis Factor (TNF) exposure ex vivo, contrasting six newly diagnosed JIA patients with two healthy controls. TNF is a pro-inflammatory cytokine that has a recognized role in the pathogenesis of disease and is the target of frontline biologic therapy in JIA as well as in several other autoimmune conditions [28]. Although the amount of TNF present in synovial fluid is not predictive of patient response to TNF inhibition [29], it may be possible to identify a subset of responders based on differential transcriptional response to TNF stimulation. Our experiments were under-powered to reveal clinically meaningful inter-individual differences, but do demonstrate cell-type specific differences in TNF response and highlight heterogeneity of case–control comparisons. To overcome sparsity of single cell data for estimation of the influences of technical and biological sources of variation [30, 31], we also introduce “scPool”, an iterative pseudo-bulk analytical pipeline for single cell analysis. We conclude that monocytes are more transcriptionally affected by TNF stimulus than lymphocytes and that memory B and T cells show mild JIA effects on gene expression that are not seen in the naïve counterparts.

## Methods

### Study subjects

Patients were recruited from the Pediatric Rheumatology clinic at Emory Children’s Center in Atlanta, GA. Patients were eligible if they (1) were diagnosed with oligoarticular or polyarticular JIA according to ILAR criteria within the past six months, (2) did not have any additional autoimmune or autoinflammatory comorbidities, and (3) were not yet prescribed biologic or DMARD therapies. Administration of NSAIDs was not criteria for exclusion. Controls included two healthy adults under the age of 25 years without any medical conditions or family history of autoimmune disease. Blood samples were collected from patients receiving routine labs in the clinical laboratory. Healthy Control patients received a CBC in the research laboratory. All children in the study provided assent and parental signed consent. Controls also provided informed consent under protocols approved by the Emory IRB.

Patients and their families provided a global assessment score and completed the Childhood Health Assessment Questionnaire (CHAQ), a validated tool used to assess functional status in patients with pediatric illnesses including JIA [32]. Clinical charts were reviewed to record number of affected joints (defined as swelling or limited range of motion and tenderness), the presence or absence of uveitis, the presence of rheumatoid nodules, and the physician’s global assessment. Laboratory values including a CBC, ESR, CRP, ANA, RF, and anti-CCP were recorded. To evaluate disease activity, JADAS-27 [33], a validated disease activity tool for JIA that includes active joint count, physician global assessment, patient global assessment, and inflammatory marker ESR, was used. All patient data was stored in a Redcap database maintained at Emory University.

### Cell preparation

Whole blood was collected from research subjects into 3 mL lithium heparin vacuum containers. Baseline cell counts of WBCs in whole blood were obtained via laboratory CBCs. Under a culture hood, whole blood was then gently transferred via syringe needle (26 3/8 G) into two 1 mL TruCulture whole-blood culture tubes, one supplemented with tumor necrosis factor alpha (TNF-α, 10 ng/ml) (Part # 782–001,295) and one negative control (NegCo) tube (Part # 782–001,291), both types purchased from Myriad RBM (Austin, TX). After 24 h ex-vivo stimulation at 37 °C, insertion of a valve separator yielded cell pellets. Post-stimulation cell counts and laboratory CBCs were performed on all samples at this time to assess cellular viability and to characterize cell population proportions.

Following stimulation, samples were washed with custom RPMI-1640 deficient in biotin, L-glutamine, phenol red, riboflavin, and sodium bicarbonate (def-RPMI-1640), and containing 3% newborn calf serum (NBCS) and spun down at 450 g for 5 min at 4 °C. 500uL of whole blood pellet was resuspended in the original whole blood volume with def-RPMI-1640 with 3% NBCS and aliquoted for PBMC isolation by negative selection using Stem Cell Technology EasySep Direct Human PBMC isolation kit (catalog #19,654), per manufacturer’s instructions and yield counted (Cellometer K2, Nexcelom).

### Single cell RNA sequencing

PBMCs were passed through a 70 µm filter, washed twice in defRPMI-1640 with 0.04% bovine serum albumin (BSA). PBMCs were resuspended at 1200 × 10^6^ cells/mL in def-RPMI-1640 with 0.04% BSA and passed through a 20-µm filter followed by cell count to confirm cell concentration. Single cell suspensions were then loaded into a Chromium Single-cell Controller (P/N 110,211) in accordance with 10X Genomics recommendations for a target cell recovery of 3000 cells per sample. Single-cell gene expression libraries were prepared using Chromium™ Single Cell 3' Library & Gel Bead Kit v2 following manufacturer’s specifications (P/N 120,237, 10X Genomics). cDNA library sequencing was performed on a NextSeq 550 at the Georgia Institute of Technology Molecular Evolution Core in 4 batches (4 samples/batch). Batches 1 and 2 were run on a Flowcell v2.0 while batches 3 and 4 were run on a Flowcell v2.5.

### Cell clustering

Sequence data were run through the Cell ranger v2.2.0 pipeline as per manufacturer guidelines. Default parameters were used without additional filtering. Subsequently the Seurat R Package (v1) [34] was used for standard preprocessing and downstream analysis. For quality control, cells were filtered manually based on QC metrics. Cells with percentages of mitochondrial genes > 15% or those with a clear outlier number of genes (> 3 SD) were removed as possible multiplets. Initially, we performed clustering on each batch separately. The global scaling method LogNormalize was then applied to normalize gene expression measurement for each cell by total gene expression. Highly variable genes were then identified for anchoring integration, using the variance stabilizing transformation option to find 3000 genes. Principal component analysis identified 32 PC and uniform manifold approximation and projection was performed with canonical correlation analysis used to find clusters at resolutions of 0.4, 0.6, 0.8, 1.0 and 1.5. We determined 17 clusters as the optimal number for all downstream analysis as this balanced resolution and repeatability. More information regarding batch comparison are detailed in the Supplemental Methods in Additional File 1.

### scPool and Variance partitioning

The *scPool* strategy was implemented using custom R scripts to assign cells within each sample and cluster to pseudocells of size 5, 10, 15 or 20 cells. As many pseudocells were constructed as the number of cells allowed: for example, if there were 63 cells in a particular cell type for a specific donor, then this supported generation of 12, 6, 4, and 3 pseudocells of the indicated sizes, respectively. The process was then iterated three times to generate pseudo-replicates for cross-validation. Only 5 or 10 cell pseudocells could be generated for the rarer cell types such as macrophages and dendritic cells. Once cells were assigned to a pseudocell, all of the counts for each gene were summed, and then these values were converted to counts per 100,000 simply by dividing by the total number of counts and multiplying by 100,000.

Variance components were assessed using *variancePartition* software [35], treating Stimulus (TNF or Control), Disease Status (JIA or Healthy Control), Batch (1, 2, 3 or 4), and Individual donor, as well as the Interaction between Stimulus and Status, as random effects. The reported values are for all genes with an average of 1 or more reads per 100,000 across the pseudocells of a cell-type, which was typically ~ 10,000 to ~ 13,000 genes per cell type. This yields an overall average variance explained per gene, as well as a list of the per-gene variance component for the cell-type. To evaluate the concordance of these highly differentially expressed genes, we evaluated the area under the concordance curve (AUCC) [36] by taking the top 100 genes for the variance component (e.g. Stimulus effect in Memory T cells) and contrasting these with the top 100 genes for another set (e.g. Stimulus effect in Naïve T cells) by computing the number of features in common in the rank order from 1 to 100 genes. The ratio of the area under this curve to the maximum possible feature sharing (100^2^/2) is the AUCC metric, which ranges from 0 to 1, and was typically over 0.95 for replicate pseudocell iterations of the same cell-type and effect.

### Pathway analysis

Pathway enrichment in the lists of top 100 variance component genes was assessed using the ToppGene suite [37] which implements a version of gene ontology analysis based on an excess of genes in a particular annotated set or pathway relative to all genes in the genome. Additional information regarding evaluation of IL-2 related genes are given in the Supplemental Methods in Additional File 1, JIA_Imbach_supplement_20.

## Results

### Subjects

The 8 individuals included 6 children with JIA (4 female) and 2 young adult female healthy controls (see Table 1). Four of the cases had oligoarticular-JIA and two had polyarticular, RF negative JIA. One of the subjects with polyarticular JIA and one with oligoarticular JIA had positive CCP antibodies but negative rheumatoid factor. The active joint counts, physician global and patient/family global assessment and other detailed participant attributes are shown in Table 1.

**Table 1:** Clinical Characteristics of Subjects with JIA

	J-11	J-12	J-13	J-14	J-15	J-17
Sex	F	F	M	F	F	F
Race	White	White	White	White	Asian	Asian
Age of onset	2	15	12	1	7	5
JIA category	Oligo	Poly	Oligo	Poly	Oligo	Oligo
Joint Count	1	18	2	17	1	1
Patient global assessment	4	9.5	4	4	1	2
Physician global assessment	3	6	6	9	2	2
CHAQ	1.12	2.30	0.25	2.12	0	2
JADAS-27	8	33.5	16.5	30.9	5	9
ANA	Negative	1:320	Negative	Negative	1:320	Negative
RF	ND	Negative	Negative	Negative	Negative	Negative
CCP status	Negative	> 250	Negative	Negative	80	Negative
B27	ND	Negative	Negative	Negative	Negative	Negative
Medications	Naproxen	Naproxen	None	Allergy med^*^	None	None

Subject J-12 also had a diagnosis of cerebral palsy. Subject J-13 developed Crohn disease several months after diagnosis of arthritis. Subjects J-11 and J-12 were on a nonsteroidal anti-inflammatory drug (Naproxen) at baseline, but none of the others were on any medication to treat their joint symptoms. None of the subjects had received glucocorticoid drugs. Subject J-14 reported being on albuterol inhalers and intranasal fluticasone

Healthy control C14 was a 22-year-old non-Hispanic white female and C22 was a 25-year-old Asian female. Neither reported a history of autoimmune diseases or being on any medications at the time of participation

### Cell proportions by disease status and stimulus

Peripheral blood samples were collected once from 8 individuals including 6 children with JIA and 2 young adult female healthy controls (Table 1, for participant attributes). For each participant, separate 1-mL cultures of peripheral blood were incubated with or without TNF activation for 24 h at 37 °C, after which an aliquot was used for single cell RNA sequencing on the 10X Genomics Chromium platform.

**Fig. 1:**
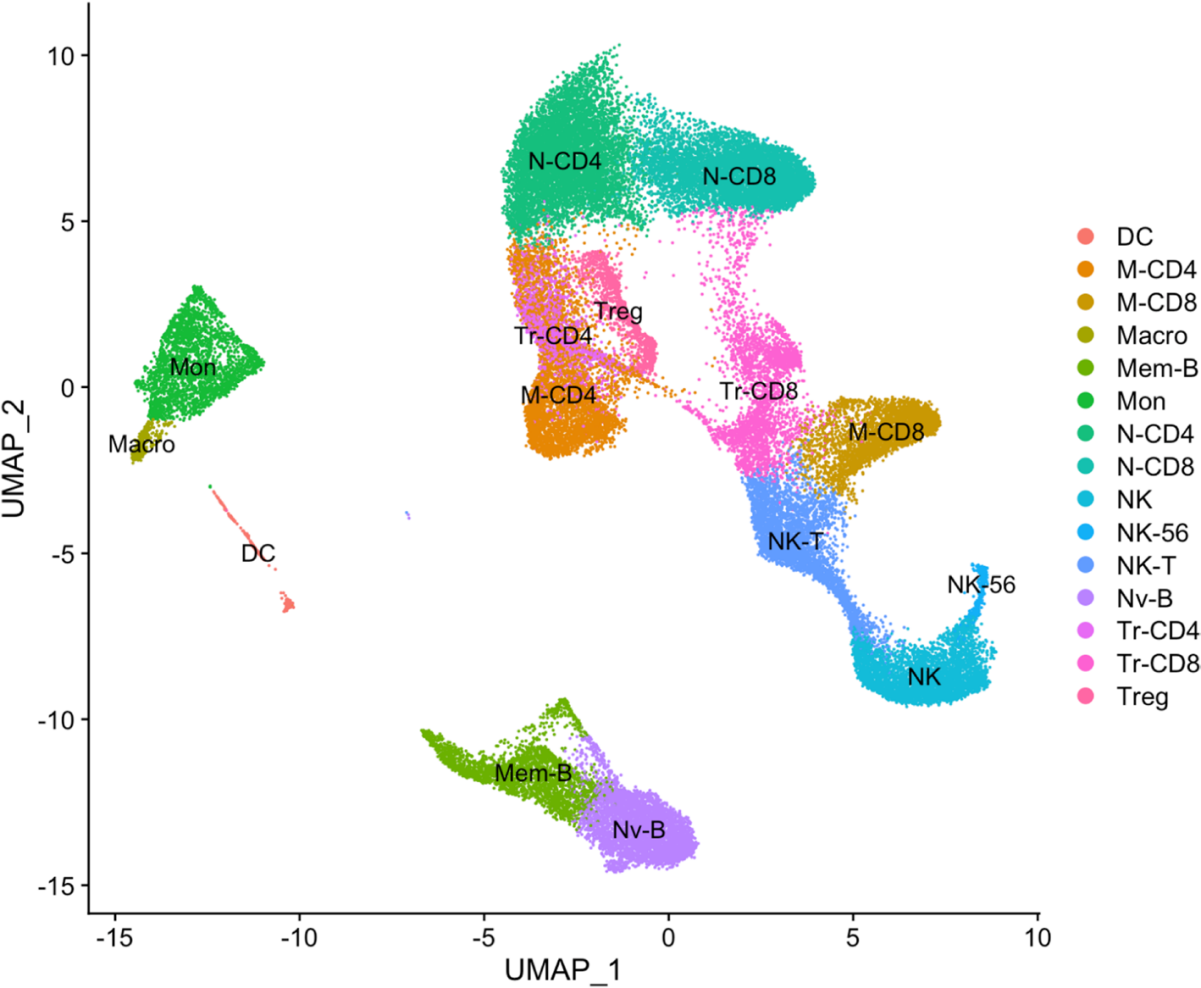
UMAP Projection of Single Cell Clusters. The projection shows the first two UMAP dimensions for all 45,943 PBMC cells across 16 samples, colored by clusters representing the indicated cell types. 3 additional clusters of cell states are partially hidden within the T-cell component. UMAP scores were computed in Seurat^45^

Across the 16 samples, after quality control we analyzed a total of 45,943 single cells, an average of just under 2,900 per sample. Using the integrated analysis pipeline in Seurat with cluster-specific anchors, we identified 17 clusters of cells whose proportions ranged from 0.8% (macrophages) to 15% (naïve CD4 + cells) of the total sample. As described in the Supplementary Data S1, cells were only included in this analysis if they were assigned to the same cluster in at least two of four batch comparisons as well as in the total dataset, ensuring that downstream analyses rely on reproducible cell assignments. Approximately 7.3% of all cells (3,629 of 49,572) did not meet this criterion and were excluded from the final dataset. The 17 cell-types, 14 of which are visible in the UMAP projection in Fig. 1 (the other 3 are embedded within the main T cell region), were assigned likely identities by matching key marker genes to the literature, resulting in 9 T-cell populations (naïve, memory, and transitional CD4 + and CD8 + cells as well as T_reg_ and two other clusters), 3 NK cell-types, naïve and memory B-cells, as well as monocytes, macrophages, and monocyte-derived dendritic cells. A small percentage of cells fell into smaller clusters likely including plasmacytoid dendritic cells and doublets, which were also excluded from further analysis. UMAP projection of all cell types retained for analysis are also depicted on a per-individual basis in Supplemental Fig. 2.

**Fig. 2:**
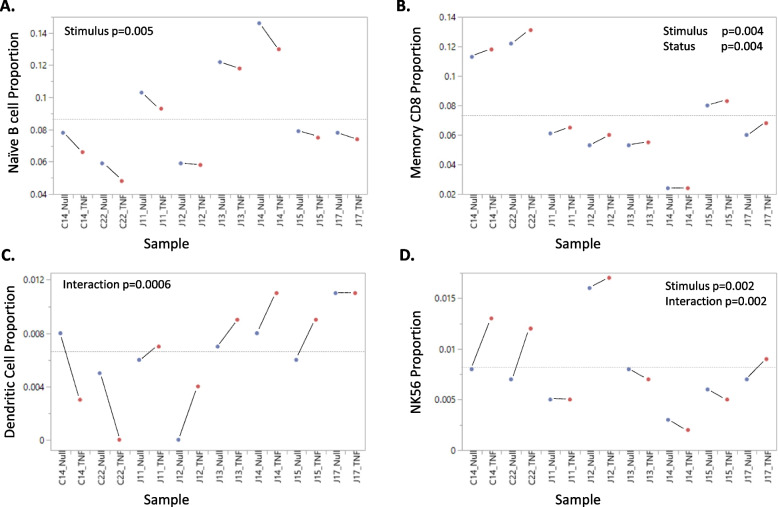
Notable influences of Stimulus and Status on Cell Proportions. Each plot shows the proportion of the indicated cell type in the total cell population for each sample, ordered in pairs of Null (unstimulated) and TNF (stimulated) with the two healthy controls on the left and six JIA to the right. The prefixes C and J in sample names represent Control and JIA respectively. Significant effects by mixed novel ANOVA of the proportions are indicated (non-significant otherwise), including Stimulus for naïve B-cells, CD8 + T-cells, and NK56 cells; JIA Status for Memory CD8 + T-cells; and the Stimulus × Status interaction for Dendritic and NK56 cells. These effects can be seen in the slopes of the lines connecting each pair of samples

In order to evaluate the influences of TNF stimulus and JIA case–control status on cell abundance, we next fit a linear mixed model to the cell type proportions within individual samples. The model evaluated fixed effects of Stimulus, Status, and the Interaction between Stimulus and Status, conditioned upon random effects of Donor and Batch (two donors in each of 4 batches). Significant effects of Stimulus were seen for five cell types (compared with one expected by chance), notably with elevation of memory CD8 + T-cells and NK56 cells, but down-regulation of naïve B-cell proportions, after exposure to TNF. Memory CD8 + and CD4 + T-cells were also both significantly elevated in the two controls relative to JIA cases. Figure 2 shows these proportions by Donor and Stimulus, including two examples of a highly significant interaction effect: dendritic cell proportions decreased markedly in the controls but increased in the JIA cases after stimulus, while NK56 cell proportions showed the opposite response. Supplementary Table S2 in Additional File 1 presents, for each of the 17 cell types, the F-ratios and p-values for each effect along with the percentage of PBMC and the proportion of variance explained by Donor differences. The Donor variance component explains most of the variance for all but macrophages, implying that among individual variation in cell proportions is large relative to influences of disease Status and Stimulus.

### Variance components of gene expression by disease status and TNF stimulus using scPool

Most existing methods for estimating differential expression in scRNAseq data fail to account for among individual variation, which is complicated by the high zero counts in the data. They also inflate significance estimates by treating single cells as biological replicates, rather than as technical replicates. Collapsing of single cell transcript counts into pseudo-cells for each individual generates transcriptome profiles that more closely resemble bulk RNAseq data and hence are suitable for variance partitioning by fitting mixed models that adjust for random effects of individual and technical batch, while also supporting robust inference of interaction effects. However, having just two healthy controls in this dataset greatly reduces the power for the disease Status comparison. In order to overcome these limitations, we devised a compromise analytical workflow, *scPool*, in which we create multiple small pools of cells of each cluster and individual, then analyze these as pseudo-cells using *variancePartition* [35] software in R to partition the sources of variance.

The repeatability of inferences from *scPool* was validated by contrasting the results when using 5, 10, 15, or 20 cells per pool. Smaller pools allow for more pseudo-cells per individual, increasing statistical power in linear modeling, while larger pools have fewer zero counts in the pseudo-cells which should increase the proportion of genes with measurable fixed effects. For each set of pools, we randomly assigned cells to pseudo-cells 3 times to evaluate the repeatability of the procedure. Of the 17 cell types, we focus on 9 which have enough cells in each individual to generate multiple pseudo-cells, and which are well-recognized immune sub-types: memory and naïve CD4 + and CD8 + T-cells, NK-T, NK, memory and naïve B-cells, and monocytes.

**Fig. 3:**
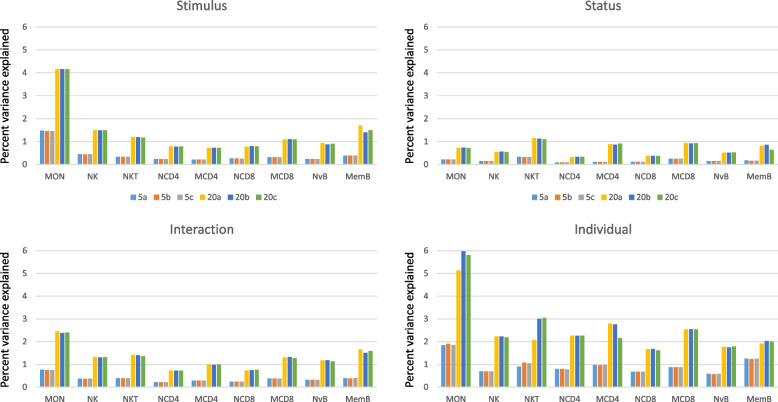
Variance components of gene expression. Bar plots show the average proportion of variance explained across all expressed genes by Stimulus, Status, Interaction, and Individual donor terms, assessed using *variancePartition* software, for the 9 indicated cell types. Colors represent three replicates of 5-cell and 20-cell pseudo-cell pooling assignments in *scPool*, clearly showing high repeatability and increased variance explained as more cells are pooled. Individual has the largest overall effect, followed by TNF stimulus

The mean proportion of variance explained by Status, Stimulus, their Interaction, or Individual differences for the 3 replicates of 5 and 20 pseudo-cell scPool datasets are shown in Fig. 3. Although the proportions are low, this is across all genes expressed in each cell type (11,000–13,000), most of which are invariant. The measure is highly correlated with the proportion of genes for which the effect explains more than 5% of the variance but has higher repeatability. We emphasize three main conclusions.

First, increasing the pool size from 5 to 20 cells typically increases the variance explained between two- and threefold, consistent with the intuition that more genes contribute to the estimate. Repeatability across the replicates is extremely high. Second, the variance contributions to gene expression within cell types is greatest for Individual differences, then TNF-Stimulus, with only a very small effect of disease Status – in fact, the Interaction between status and stimulus explained slightly more variance. This result implies that among individual differences, even after adjusting for the batch effect of processing individuals in pairs, contribute to overall gene expression profiles as well as cell proportions. Third, monocyte gene expression is two and a half times more influenced by Individual and Stimulus effects than any of the other cell types. Disease Status did not meaningfully influence monocyte gene expression, but memory B, and CD4 + and CD8 + T cells were all more differentiated between JIA patients and controls than their naïve counterparts. This result provides *prima facie* evidence that JIA is accompanied by changes in gene expression as B and T cells differentiate, whereas there are no significant intrinsic differences in the naïve cells.

### Differential gene expression by disease status and TNF stimulus

Next, we asked how consistent the gene expression differences were among cell types as a function of Status and Stimulus, by computing the AUCC for the lists of genes with the top 100 variance components of each effect [36]. Supplemental Fig. 2 summarizes the concordance among cell types using the pseudo-cell matrices generated with 20 cells; similar results were seen with 5-cell matrices. Once again, TNF-stimulation had a much more consistent effect on gene expression profiles than disease Status. Approximately 90% of the genes are in common in the three replicate sets of 100 most differentially expressed genes for T cells or for Monocytes, but unexpectedly this repeatability dropped to the range of 50% to 60% for the B cell subsets, implying greater variability in their response to stimulus. The four T cell subtypes all show fairly high concordance with one another, close to 50%, which drops somewhat in the NK and NK-T cells, implying that they respond differently to stimulation. Monocytes and B cells had very low concordance, also implying a different response to TNF-stimulation. With regard to disease Status, concordance of the four T cells subsets was low, of the order of 20%. Intriguingly, NK cells overall had more similarly differentially expressed genes by JIA Status in common with T cells than did NK-T or B cells, and Monocytes had no commonality with the other cells.

Gene ontology analysis [37] indicates that TNF induces the expression of over 50 cytokine response genes (GO:0,034,097), as well as transcripts encoding proteins related to cell–cell adhesion (including 15 cadherin-binding proteins (GO:0,045,296), and cysteine-type peptidases (GO:0,008,234), but suppresses 12 genes related to lymphopenia (HP:0,001,888). The most uniformly and strongly induced genes include three components of NF-κB signaling (*RELB*, *NFKB2*, and the inhibitor *NFKBIA*), the interleukin *IL32* (an activator of TNF from macrophages) and interleukin receptor *IL2RG*, *LTB* lymphotoxin, and immune cell antigens *CD7* and *CD74*. The three most uniformly down-regulated genes encode the thioredoxin binding protein *TXNIP*, a transcription factor involved in inflammation and leukocyte viability, *KLF2*, and the leukemia antigen *CD52*. A set of 70 genes more strongly affected by stimulus in myeloid cells is highly significantly enriched for 17 genes annotated to myeloid-mediated immunity (GO:0,002,444) that are also engaged in secretory vesicle function or production (GO:0,099,503). In addition, ribosomal proteins are relatively up-regulated, specifically in TNF-exposed Memory T-cells and NK cells, but down-regulated in naïve B-cells, monocytes, and macrophages. By contrast, there was relatively little overlap among cell types in the identities of the genes differentially expressed with respect to disease Status.

### Cell-specific induction of interleukin response pathways

The IL-2 pathway has a critical role in T-cell activation and development. In addition, this pathway has a key role in the maintenance of immune tolerance through the dependence of regulatory T-cells on IL-2. Associations between JIA and *IL2* and *IL2RA* variants reported in RA had been replicated in JIA [38]. The *IL2RA*, *IL2-IL21* and *IL2RB* loci all demonstrated genome wide levels of significance in the Immunochip study of JIA, the largest multinational collaborative cohort study of JIA with 2816 JIA cases [39]. Two other loci confirmed to be associated with JIA also play a role in the IL-2 pathway: *SH2B3* encodes an adapter protein involved in T cell activation whilst *STAT4* encodes a transcription factor critical for T cell differentiation. Together these associations support the role of the IL-2 pathway in JIA susceptibility. Given this, we evaluated whether the IL-2 pathway may have been associated with disease status, independently or in connection to TNF stimulation, in our dataset. Evaluating scaled expression of genes associated with IL-2 stimulation and repression was conducted per sample within each cell type [40]. No clear patterns were observed with JIA status. The only clear indication of gene influence by disease or stimulus was the *LTB* signature observed in TNF-stimulated T cell fractions (Supplemental Fig. 3, Additional File 1). *LTB* is a member of the TNF family of genes and has been well described in inflammation responses but does not clearly indicate any bias toward JIA status here.

**Fig. 4:**
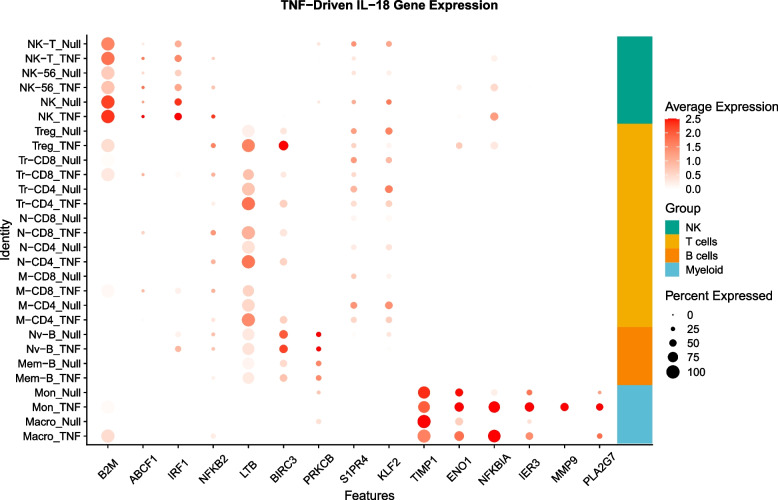
Dot plots showing variability of expression of IL-18 pathway genes. Each dot shows the average expression (log2 counts per 100,000 of positive cells) of the indicated gene (columns), with the size of the dot scaled to the proportion of cells positive for the gene. Darker red is higher expression. Genes are clustered from top to bottom in sets of NK cells, T-cells, B-cells, and Myeloid cells, with Null above TNF-stimulated in each pair. Even though the IL-18 pathway is engaged by stimulus in each cell type, different subsets of genes are responsible for the response

From the perspective of RA pathology, a particularly interesting pathway is IL-18 signaling, which has been implicated as a proinflammatory mediator of T_helper_ cell activation via a positive feedback loop with TNF in the synovium [41]. Genes annotated to the KEGG IL-18 pathway were highly significantly enriched in the top 100 stimulus effect genes in multiple cell types in our dataset, but with only partially overlapping gene sets. Figure 4 shows how 15 IL-18 response genes form four clusters with respect to PBMC cell-type specific expression: *B2M* and *IRF1* are exemplars of natural killer cell TNF induction, *LTB* and *BIRC3* of T-cell induction (intriguingly, *S1PR4* and *KLF2* are down-regulated in T-cells), *PRKCB* is somewhat B-cell specific, and *TIMP1*, *ENO1*, and *NFKBIA* are all myeloid responsive only. Another pathway showing similar cell-type specific activation of different members was actin-related genes, suggesting variable effects of TNF on the cytoskeleton.

### Engagement of the HLA complex in JIA cases

**Fig. 5:**
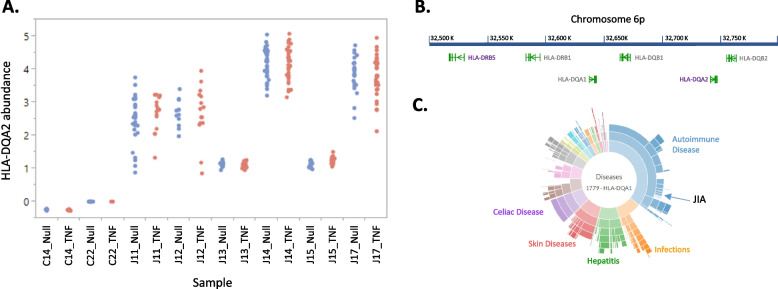
HLA expression in JIA patients. (**A**) HLA-DQA2 gene expression in pseudo-cells is suppressed in both healthy controls (left) but elevated to variable degrees in the JIA cases, with no effect of TNF stimulus. (**B**) Schematic map of the 300 kb region of the HLA encompassing HLA-DQA2 and HLA-DRB5 showing locations of 4 other HLA genes not differentially expressed by disease Status. Drawn from UCSC browser annotation, vertical bars represent exons and arrowheads direction of transcription. (**C**) Sunburst plot of documented disease associations with SNP variation in the vicinity of HLA-DQA1, highlighting JIA, plotted with HLA-spread (http://hla-spread.igib.res.in) [42]. Rings indicate increasing levels of haplotype resolution and each ray is a disease sub-type within the broader category

Genome-wide association studies have associated variation in the HLA complex with JIA through the regulatory polymorphism rs7775055-G [39]. We thus examined whether expression of HLA complex genes is altered in specific immune cell types. Two HLA loci, *HLA-DQA2* and *HLA-DRB5*, are by far the most up-regulated genes in both Naïve and Memory B-cells, as well as in all three myeloid cell types (monocytes, macrophages, and dendritic cells) as shown in Fig. 5. Although neither gene is detectable in the two Control donor samples, they are robustly expressed in the five antigen-presenting cell types in all six JIA samples, both with and without TNF stimulus, but have low expression in T-cells. Neither *HLA-DQA2* nor *HLA-DRB5* have been associated with JIA by GWAS. They are located 220 kb apart in the complex, separated by three other HLA-D regions genes, so co-regulation in *cis* would entail long-range regulation. Polymorphism near *HLA-DQA2* has previously been associated with mild influenza (H1N1) susceptibility, whereas *HLA-DRB5* associates with RA, white blood cell counts, and a dozen other traits. Notably, Wu et al. [26] show elevated abundance of HLA-DRB5 + plasma cells in RA patient peripheral blood relative to controls, but note that this may be biased toward ACPA-positive cases in the synovial tissue.

## Discussion

This study was conceived as pilot investigation into the potential of single cell profiling along with ex vivo TNF stimulation to illuminate the cellular basis of personalized immune responses in JIA. Personalized immunotranscriptomics has been implemented in the context of SLE where longitudinal bulk RNAseq implicated half a dozen different modes of disease progression (4), which were further characterized by single cell peripheral blood profiling in 33 children with the disease (5). We have similarly used bulk immune profiling to observe multiple different pathways of resolution of complicated malaria [43]. Multiple eQTL studies have demonstrated that the genetic regulation of gene expression is meaningfully modified by ex vivo stimulation with a range of stimulants [44–47], implying that personalized immunoprofiling may benefit from comparison across culture conditions. Our results are consistent with this conclusion since we document significant individual-by-TNF stimulus interactions in terms of both cell-type abundance and gene expression.

In fact, we found that cell differences were more pronounced between experimental treatment groups than those observed between JIA patients and control subjects. The concept that immune cell states converge in the presence of similar inflammatory signals across different broad disease phenotypes is not a novel observation. A study harnessing scRNAseq analyses to investigate immune states in COVID-19 and other inflammatory diseases found that a TNF driven macrophage state was observed in in severe COVD-19 and inflamed rheumatoid arthritis synovium when compared to non-inflamed rheumatoid arthritis and non-inflammatory osteoarthritis. [48] This finding supports the notion that a particular immune stimulation may drive immune states across multiple inflammatory diseases, and perhaps similarly explains the stronger discrepancy in this study between TNF stimulated and unstimulated samples. However, much larger sample sizes will be required to elucidate whether this approach either demonstrates correspondence between immune profiles and JIA sub-types or illuminates novel molecular sub-types of disease.

We chose TNF as the stimulant because of the efficacy of anti-TNF therapies for the treatment of JIA, RA and other autoimmune diseases. A caveat to our analysis is that some of the effects are likely mediated by the influence of secondary cytokine secretion as well as cell death particularly of neutrophils in culture, yet we clearly observe activation and repression of distinct sets of transcripts in T-cells and myeloid cells, with a dampened response in B-cells. It is impossible to know to what extent the dominant effect of stimulation in our study compares with the effect of inter-individual differences in TNF levels or of clinical inhibition of TNF. However, we note that changes in cell type proportions are only of the order of a few percent, and inter-individual differences remain the major source of variance, implying that the treatment is not overwhelming. The observed differential expression involves different pathways, but also genes within pathways such as IL-18 response and actin cytoskeleton organization. The functional implications remain to be elucidated, but we note that the common practice of reporting pathway activation scores based on KEGG and other databases is likely a crude representation of actual cell-type specific responses. Upregulation of the IL-2-pathway suggests a role for anti-T cell strategies such as JAK-STAT inhibitors or cyclosporine in some refractory polyarticular/oligoarticular JIA patients. While the role of IL-18 pathway in systemic JIA is recognized, confirmation of IL-18 pathway involvement may suggest that some children with oligoarticular/polyarticular JIA are candidates for IL-1 targeted therapies.

Another novel aspect of this study is the introduction of the *scPool* strategy for quantifying variance components in a complex experimental design. The motivation is that existing methods for accounting for random effects in single cell data, such as MAST [49], are mostly used to adjust for batch effects rather than individual differences, and it is not known how well they capture interaction effects. Implementation of *scPool* could follow two main strategies. One, as demonstrated here, is to define a constant number of cells per pseudo-cell across all samples, which can be varied for example from 5 to 20, allowing ascertainment of the repeatability of variance component inference. An advantage is that all cell-types are pooled equivalently, but a disadvantage is that less abundant cell-types either have fewer pseudo-cells or even drop out of the analysis. Alternatively, the decision could be made to generate the same number of pseudo-cells for each cell-type and individual, which ensures that statistical power is conserved across cell-types, but means that rarer cell-types may only have a few cells per pseudo-cells whereas very common ones may have one hundred or more. This inevitably also alters power at the level of individual genes which vary widely in abundance. Our recommendation is to compare a variety of strategies for consistency, focusing more on relative contributions to the variance than p-values per se, and to correlate resulting pooled counts to sample-matched bulk RNA counts.

A major limitation of this study is the small sample size, which is insufficient to document to what extent individual donor differences in their baseline and TNF-responsive immune profiles correlate with clinical features of JIA. This is particularly true given that the modest number of individuals analyzed herein displayed heterogeneous disease phenotypes within the general scope of JIA. Specifically, two of the six JIA patients were CCP positive, but we did not have statistical power to evaluate the influence of this condition on the differential cell abundance or gene expression between JIA cases and control subjects. In future studies, we recommend increasing sample size and ensuring that, if a heterogeneous patient population is included, there are sufficient patients of each disease sub-type to permit specific comparisons of each with one another and/or with included control subjects. Even samples of 100 patients may not be sufficient to this end, but in an era where GWAS often exceeds one million participants, the potential for individualized functional profiles to guide therapeutic choices warrants further pursuit of single cell immunotranscriptomics. Another limitation is ongoing uncertainty about cell-type assignments from single cell data. We detect all of the immune cell-types automated in the new Seurat Azimuth pipeline [50], but also find subsets of intermediate T-cells of uncertain identity or state. Partitioning of sub-sets of NK cells, B-cells, and rarer myeloid cells also follows standard current practice, but it is important to note that inferences regarding proportions of these sub-types may be influenced by choice of parameters in the clustering algorithm.

## Conclusion

In conclusion, we show here that 24 h ex vivo TNF stimulation influences differentiation of multiple arms of the peripheral blood immune compartment to varying degrees, superimposed on individual differences that are likely to relate to variable therapeutic responses for children with idiopathic arthritis. There are hints of intrinsic differences in gene expression in JIA cases as well as of an altered profile of response to stimulation. Larger studies of this nature have the promise to illuminate mechanisms of treatment response and to guide therapeutic intervention for diverse immune diseases.

## Supplementary Information

**Additional file 1.**

## Data Availability

The datasets generated and analyzed during the current study are available in the Gene Expression Omnibus repository as GSE205095.

## Abbreviations

JIA: Juvenile Idiopathic Arthritis
ILAR: International League of Associations for Rheumatology
RF: Rheumatoid Factor
HLA: Human Leukocyte Antigen
DMARDs: Disease Modifying Anti-Rheumatic Drugs
AUCC: Area Under the Concordance Curve
TNF: Tumor Necrosis Factor
RA: Rheumatoid Arthritis
