# Supplementary material for: Profiling the peripheral immune response to ex vivo TNF stimulation in untreated juvenile idiopathic arthritis using single cell RNA sequencing
Source: Pediatr Rheumatol Online J. 2023 Feb 15;21:17. doi: 10.1186/s12969-023-00787-x (PMC9929251; doi:10.1186/s12969-023-00787-x)
Supplement: Supplementary file 1 — Additional file 1. [file 12969_2023_787_MOESM1_ESM.docx]

**Profiling the peripheral immune response to ex vivo TNF stimulation in untreated juvenile idiopathic arthritis using scRNAseq.**

Kathleen J. Imbach^1^*, Nicole J. Treadway^2^*, Vaishali Prahalad^2^, Astrid Kosters^3^, Dalia Arafat^1^, Meixue Duan^1^, Talia Gergely^2^, Lori A. Ponder^4^, Shanmuganathan Chandrakasan^4,5^, Eliver Ghosn ^2,3,4^, Sampath Prahalad^2,4,6#^, Greg Gibson^1,4#^.

**SUPPLEMENTARY MATERIAL**

**Supplementary Table 1 Cell Type Proportions by Disease Status and Stimulus**

% PBMC^1^ Status Stimulus Interaction^2^ Donor

F ratio P val F ratio P val F ratio P val %Var^3^

Naïve CD4 15.0 2.54 0.16 1.83 0.22 0.68 0.44 92.6

Memory CD4 9.5 7.54 **0.03*** 5.10 0.06 2.53 0.16 97.8

Transition CD4 3.7 0.48 0.51 7.86 **0.03*** 0.01 0.91 97.4

Naïve CD8 13.2 1.05 0.34 0.25 0.64 6.14 **0.05*** 98.3

Memory CD8 7.1 19.89 **0.004**** 20.17 **0.004**** 1.50 0.27 98.5

Transition CD8 6.6 3.42 0.11 4.62 0.08 1.15 0.32 86.3

Treg 2.2 0.38 0.56 2.42 0.17 1.41 0.28 96.6

Low T 1.8 0.19 0.68 1.82 0.23 0.26 0.63 96.4

Other T 2.4 0.49 0.51 0.01 0.95 0.12 0.74 79.5

Naïve B 8.7 1.95 0.21 19.18 **0.005**** 1.48 0.27 98.4

Memory B 6.1 0.67 0.44 0.31 0.60 0.56 0.48 93.7

NK 8.4 0.43 0.54 0.12 0.74 0.05 0.83 95.7

NK-T 7.4 0.00 0.97 0.68 0.44 0.98 0.36 99.1

NK56 0.8 0.48 0.51 28.13 **0.002**** 28.13 **0.002**** 96.6

Monocyte 5.5 0.19 0.68 11.54 0.01* 0.25 0.64 85.2

Macrophage 0.8 0.41 0.55 2.21 0.19 0.10 0.76 53.6

Dendritic 0.7 2.01 0.21 6.67 0.04 42.67 **0.0006**** 90.1

^1^ The percentage of all PBMC represented by the indicated cell type

^2^ The F-ratio and significance of the Status×Stimulus interaction effect

^3^ The proportion of variance due to the among Donor random effect

**Supplementary Figure 1 UMAP Projection of Single Cell Clusters per Individual**


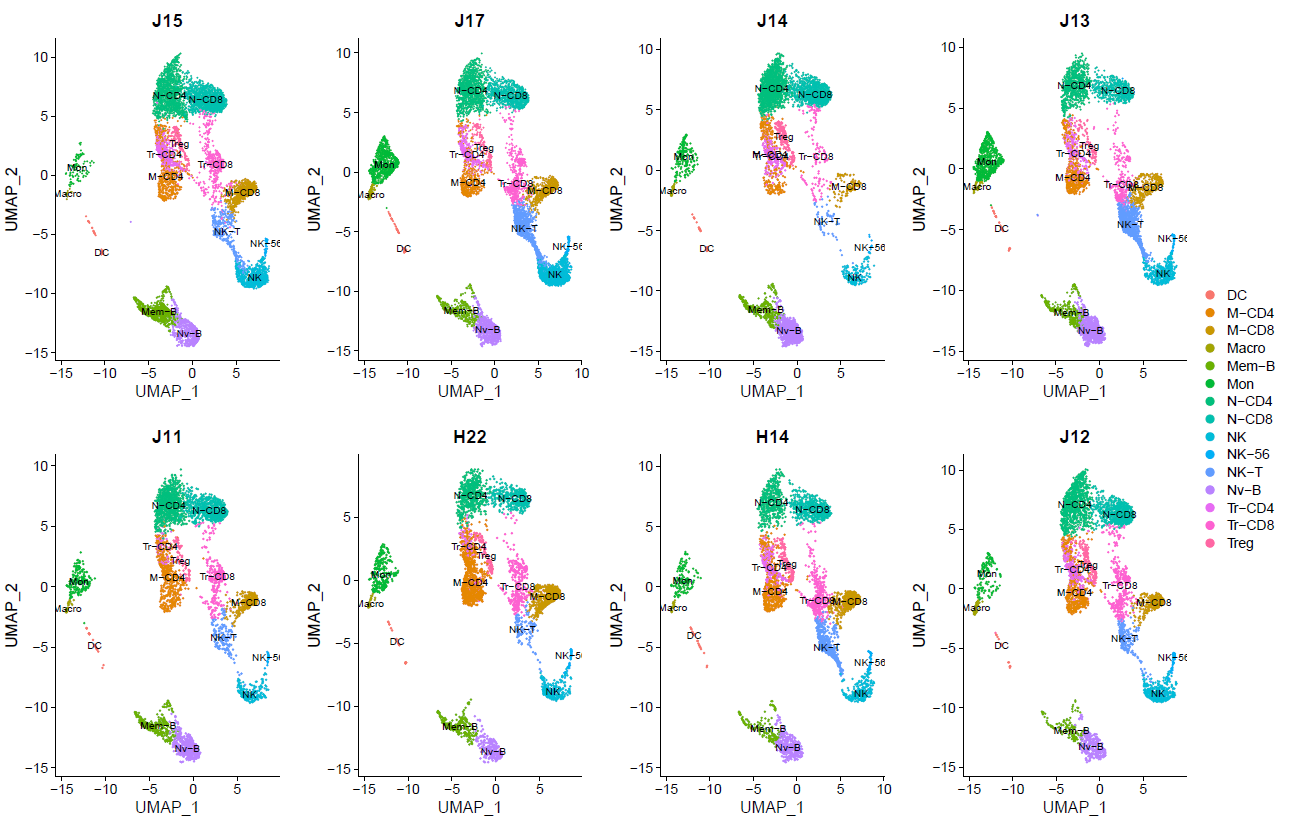


The projection shows the first two UMAP dimensions for all PBMC cells, colored by clusters representing the indicated cell types and split by individual. UMAP scores were computed in and projected with Seurat^45^. Subjects J11, J12, J13, J14, J15 and J17 indicate individuals with JIA, and subjects H14 and H22 indicate healthy controls.

**Supplementary Figure 2 Area under the concordance curve (AUCC) for differentially expressed genes**


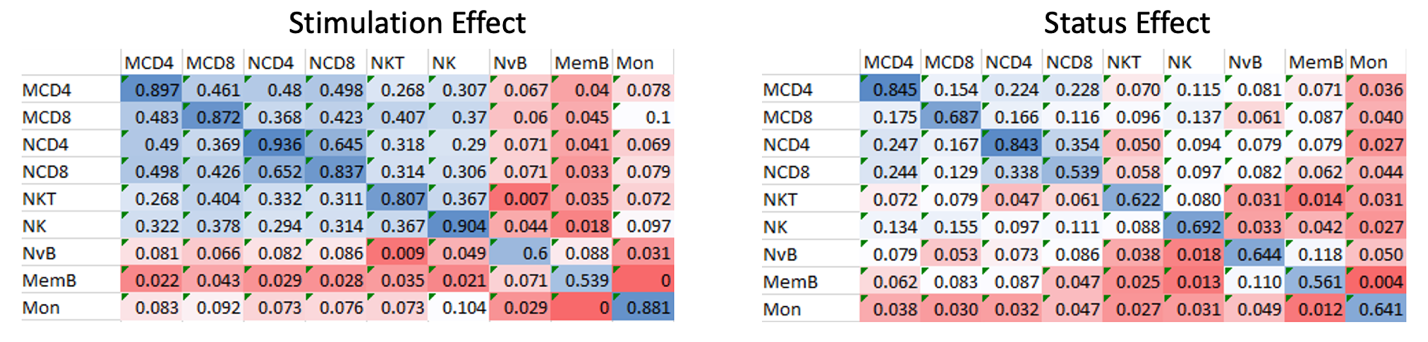


Each cell shows the average AUCC for the top 100 differentially expressed genes (highest variance component for the indicated effect) for three replicates of one cell type (columns) compared with one of the other (rows). Red tint implies low concordance, and blue high concordance. Stimulus effects are consistent across T-cell subtypes, whereas disease Status is more variable.

**Supplementary Figure 3 Scaled Expression of IL-2 Associated Genes**


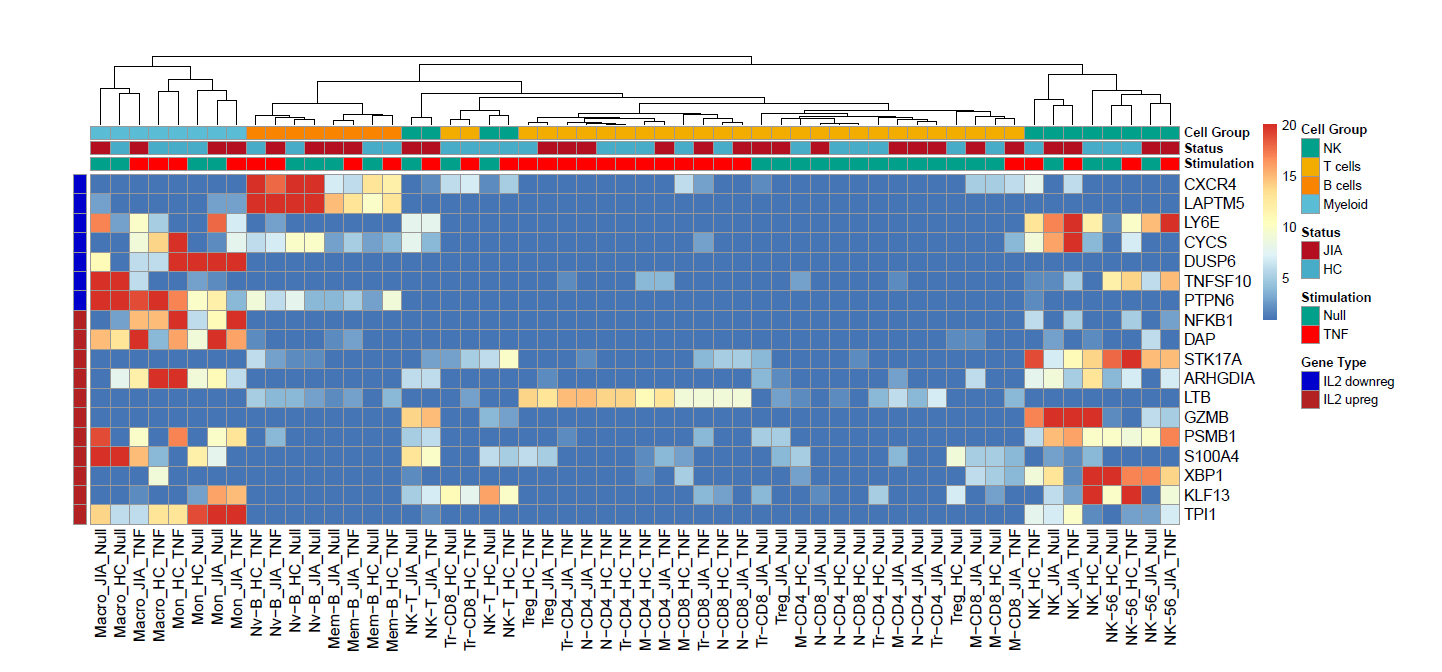


Genes associated with IL-2 stimulation (IL2 upreg) or repression (IL2 downreg) across cell types for each sample^37^. Scaled expression of each gene, generated from Seurat analysis, are shown in the heatmap. Colored bars above the heatmap indicate cell groups, sample disease status (where JIA indicates diseased subjects and HC refers to control subjects), and sample stimulation status. Colored bar to the left indicates genes’ relationship to IL-2 stimulation.

**Supplementary Data S1: Detailed Methods**

*Cell clustering*:

To assign cells to robust clusters, we reran the clustering on each pair of batches. This means that each cell was assigned to a cluster in four analyses, affording an opportunity to evaluate the concordance of cluster assignment four ways (each batch as well as each pair of batches, for example cells in batch 1 were clustered in batch 1 alone, or in pairs of batches 1 and 2, 1 and 3, or 1 and 4). The cell assignment matrices were contrasted by assessing the proportion of cells in each cluster that match to one or two clusters in another analysis, which typically led either to 80-90% of cells matching (not necessarily to the same cluster number as cluster sizes differed slightly among analyses) or to splitting/merger of cells between one or two clusters. We manually compared the concordant clusters, and required that every cell was in the same meta-cluster in at least two of the comparisons, which led to retention of 93% of the cells. The other 7% were regarded as having ambiguous identity, and discarded. Subsequently, we combined all four batches and projected the 17 metaclusters onto the UMAP projection of the first two reduced dimensions shown in Figure 1. Cell type identities were assigned on the basis of characteristic markers reported in the immune cell literature, and subsequently confirmed by reference to Azimuth^46^. A few additional T-cell types were identified, namely a population low for both CD4 and CD8, and a population with elevated Interferon response gene expression. Cell cycle status in all cells was evaluated by conducting a cell cycle module scoring approach for each cell. Because there was no significant contribution of cell cycle states according to disease status or TNF stimulation, cell cycle differences were not expected to strongly influence cell clustering or downstream expression comparisons.

*IL-2 Gene Analysis:*

Given recent associations of IL-2 signaling with JIA pathogenesis, IL-2 stimulated and repressed genes (39) were evaluated in each cell group according to disease status and treatment. This was done using Seurat’s DotPlot function, which generates scaled expression of each gene across different cell groups. The results are summarized in a heatmap for easy comparison across all groups and according to each gene’s association with IL-2 (stimulated or repressed), shown in Supplementary Figure 1.
